# Usefulness of NGS for Diagnosis of Dominant Beta-Thalassemia and Unstable Hemoglobinopathies in Five Clinical Cases

**DOI:** 10.3389/fphys.2021.628236

**Published:** 2021-02-05

**Authors:** Valeria Rizzuto, Tamara T. Koopmann, Adoración Blanco-Álvarez, Barbara Tazón-Vega, Amira Idrizovic, Cristina Díaz de Heredia, Rafael Del Orbe, Miriam Vara Pampliega, Pablo Velasco, David Beneitez, Gijs W. E. Santen, Quinten Waisfisz, Mariet Elting, Frans J. W. Smiers, Anne J. de Pagter, Jean-Louis H. Kerkhoffs, Cornelis L. Harteveld, Maria del Mar Mañú-Pereira

**Affiliations:** ^1^Translational Research in Child and Adolescent Cancer – Rare Anemia Disorders Research Laboratory, Vall d’Hebron Research Institute, ERN-EuroBloodNet Member, Barcelona, Spain; ^2^Josep Carreras Leukaemia Research Institute, Badalona, Spain; ^3^Department of Medicine, Universitat de Barcelona, Barcelona, Spain; ^4^Department of Clinical Genetics, Leiden University Medical Center, ERN-EuroBloodNet Member, Leiden, Netherlands; ^5^Hematologic Molecular Genetics Unit, Hematology Department, Hospital Universitari Vall d’Hebron, ERN-EuroBloodNet Member, Barcelona, Spain; ^6^Oncohematologic Pediatrics Department, Hospital Universitari Vall d’Hebron, ERN-EuroBloodNet Member, Barcelona, Spain; ^7^Hematology Department, Hospital Universitario Cruces, Barakaldo, Spain; ^8^Red Blood Cell Disorders Unit, Hematology Department, Hospital Universitari Vall d’Hebron, ERN-EuroBloodNet Member, Barcelona, Spain; ^9^Department of Clinical Genetics, VU Medical Center, Amsterdam, Netherlands; ^10^Department of Pediatric Hematology, Leiden University Medical Center, Leiden, Netherlands; ^11^Department of Hematology, HAGA City Hospital, The Hague, Netherlands

**Keywords:** unstable hemoglobinopathies, dominant beta-thalassemia, next generation sequencing, whole exome sequencing, rare anemia disorders

## Abstract

Unstable hemoglobinopathies (UHs) are rare anemia disorders (RADs) characterized by abnormal hemoglobin (Hb) variants with decreased stability. UHs are therefore easily precipitating, causing hemolysis and, in some cases, leading to dominant beta-thalassemia (dBTHAL). The clinical picture of UHs is highly heterogeneous, inheritance pattern is dominant, instead of recessive as in more prevalent major Hb syndromes, and may occur *de novo*. Most cases of UHs are not detected by conventional testing, therefore diagnosis requires a high index of suspicion of the treating physician. Here, we highlight the importance of next generation sequencing (NGS) methodologies for the diagnosis of patients with dBTHAL and other less severe UH variants. We present five unrelated clinical cases referred with chronic hemolytic anemia, three of them with severe blood transfusion dependent anemia. Targeted NGS analysis was performed in three cases while whole exome sequencing (WES) analysis was performed in two cases. Five different UH variants were identified correlating with patients’ clinical manifestations. Four variants were related to the beta-globin gene (Hb Bristol—Alesha, Hb Debrousse, Hb Zunyi, and the novel Hb Mokum) meanwhile one case was caused by a mutation in the alpha-globin gene leading to Hb Evans. Inclusion of alpha and beta-globin genes in routine NGS approaches for RADs has to be considered to improve diagnosis’ efficiency of RAD due to UHs. Reducing misdiagnoses and underdiagnoses of UH variants, especially of the severe forms leading to dBTHAL would also facilitate the early start of intensive or curative treatments for these patients.

## Introduction

Beta-thalassemia major (BTHAL) is a well-known life-threatening condition characterized by severe transfusion-dependent anemia. BTHAL is an autosomal recessive disorder presenting with high frequencies in populations from the Mediterranean area. Currently, up to 257 genetic variants in the beta-globin gene (*HBB*) have been identified as BTHAL disease-causing, leading to a total or partial reduction of beta-globin chain synthesis. The clinical severity of BTHAL is related to the extent of imbalance between the alpha and non-alpha-globin chains, while clinical management consists of regular life-long red blood cell (RBC) transfusions and iron chelation therapy. At present, the only definitive cure is bone marrow transplant (Efremov, 2007; Galanello and Origa, 2010). Both BTHAL patients and carriers are usually easily diagnosed through routine laboratory tests. However, there is an ultra-rare condition overlapping BTHAL clinical manifestations known as dominant beta-thalassemia (dBTHAL), which is caused by the presence of certain unstable (UH) or hyper unstable (HUH) hemoglobinopathies.

UHs are a group of congenital disorders caused by mutations in globin genes leading to destabilization of hemoglobin (Hb) molecules as a consequence of (a) amino acid substitutions within the heme pocket, (b) disruption of secondary structure, (c) substitution in the hydrophobic interior of the subunit, (d) amino acid deletions, and (e) elongation of the subunit. Thus, altering any of the steps in globin processing, including subunit folding, heme interaction, dimerization, or tetramerization (Bunn and Forget, 1986). These abnormal Hb variants undergo rapid denaturation followed by precipitation, leading to the formation of Heinz bodies, which cause hemolysis of RBCs. Clinical manifestations may vary from asymptomatic to severely affected forms. Treatment is mainly symptomatic and based on transfusion requirements as for BTHAL (Steinberg et al., 2009; Thom et al., 2013).

UHs are dominantly inherited with a significant rate of *de novo* mutations. They generally do not separate from normal Hb using standard methods. Thus, diagnosis of dBTHAL can be challenging since it requires a high index of suspicion and the diagnosis may be delayed for years hampering the access to timely treatment interventions.

The study we present herein confirms the relevance of including globin genes in next generation sequencing (NGS) approaches for the diagnosis of rare anemia disorders (RADs), especially for cases with no family history in which the anemia is not easily explained.

## Patients and Methods

### Clinical Reports

Here we present five clinical cases diagnosed with UH after NGS analysis. Clinical data and laboratory findings are shown in Table 1.

**TABLE 1:** Overview on clinical and genetic data of the five reported clinical cases.

Parameters	Case 1	Case 2	Case 3	Case 4	Case 5
**Gender/Age**	**Male/Pediatric**	**Female/Adult**	**Male/Adult**	**Male/Pediatric**	**Female/Pediatric**
Hb (120–170 g/L)	70–80	119	141	82	79
MCV (80–100 fL)	110–115	97.8	102.3	83	Not done
MCHC (27–33.5 g/dL)	28	32.1	30.8	Not done	Not done
Reticulocyte count (50–100 ⋅ 10^9/L)	900	293	331	810	Not done
Reticulocyte count (%)	34	7.71	7.39	Not done	Not done
Lactate dehydrogenase-LDH (U/L)	4,500–5,000	243	145	186	259
Hb Fractions	Normal	Normal	Normal	Not done	Not done
Heinz bodies	Positive	Negative	Positive	Not done	Not done
Stability test	Positive	Negative	Positive	Not done	Not done
Age of onset (months)	4	Unknown	Unknown	Unknown	2
Family history	No family history	Father presents mild compensated hemolysis	Mother diagnosed with hereditary spherocytosis	No family history	No family history
Transfusion need	8 U/Year	No	2 times	Multiple	Multiple
Splenectomy	Yes (5 y)	No	Yes (25 y)	No	No
Stem cell transplant (age years)	No	No	No	Yes (4 y)	Yes (3 y)
Genotype	*HBB*c.202G > A (p.Val67Met)	*HBA1*c.187G > A (p.Val62Met)	*HBB*c.290T > C (p.Leu96Pro)	*HBB*c.442T > C (p.Ter147Glnext*21)	*HBB*c.442T > A (p.Ter147Lysext*21)
Hb variant name	Hb Bristol-Alesha	Hb Evans	Hb Debrousse	Hb Zunyi	Hb Mokum

*Performed after the diagnosis of UH.

**FIGURE 1:**
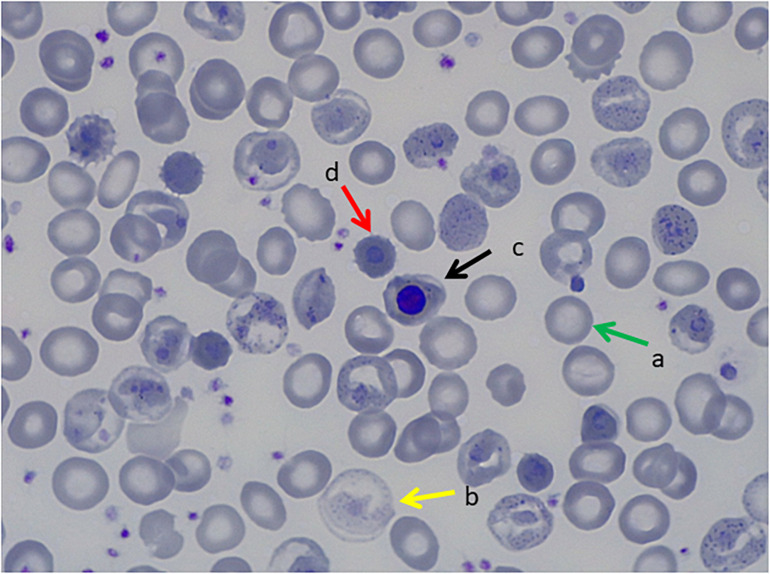
Peripheral Blood Smear, May Grunwald Giemsa Stain. **(a)** Transfused red blood cells, **(b)** non-transfused red blood cells with hemoglobinization abnormalities, **(c)** orthochromatic erythroblast, **(d)** erythrocitary inclusions that correspond to Heinz bodies.

**FIGURE 2:**
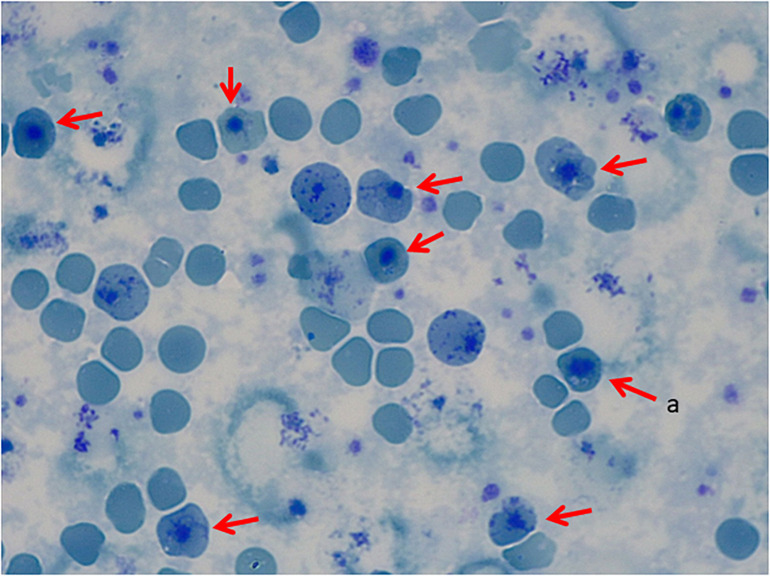
Peripheral Blood Smear, Brilliant Cresyl Blue Stain. **(a)** Heinz bodies.

**FIGURE 3:**
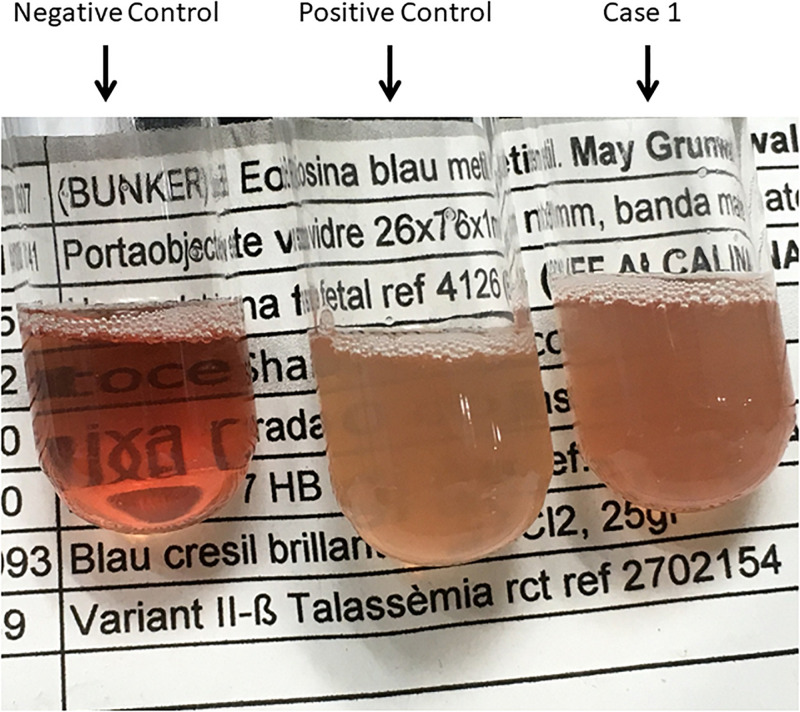
Isopropanol Test_01. Negative control (Hb AS), Positive control (Hb F), and Case 1.

The first case is a male pediatric patient referred with severe chronic blood dependent anemia since he was 4-month-old, asthenia, jaundice, and short stature. No family history of hemolytic anemia. Examination of blood smear revealed polychromasia, anisopoikilocytosis, basophil stippling, Cabot rings, schistocytes, and spherocytes. Separation and quantification of Hb fractions did not reveal any extraordinary peak and showed normal values for HbA_2_ and HbF. At 5-year-old he underwent splenectomy. After the surgery, Heinz bodies were present (Figures 1, 2) and isopropanol stability test, performed according to standard methodology, appeared positive (Figure 3). Family studies in both parents were strictly normal, including evaluation of Hb fractions. Enzyme activity assays, EMA-binding test, and osmotic gradient ectacytometry (LoRRca MaxSis) were performed to rule out hemolytic anemia due to RBC defects other than hemoglobinopathy. Results, although not strictly normal, did not reveal any RBC defect. However, they should be taken with caution since the patient was intensively transfused. Genetic analysis was performed on *PKLR* and *G6PD* genes failing to reveal any disease-causing mutation.

The second case is a female adult patient with mild chronic compensated hemolysis referred for diagnosis when she was 20 years old. The father also presented with mild compensated hemolysis. No further examinations were performed before referral. Although the presence of extravascular hemolysis, the examination of blood smear was not informative. Separation and quantification of Hb fractions did not reveal any extra peaks and Heinz body and stability tests were normal. Further laboratory tests were performed to rule out hemolytic anemia due to RBC enzyme and membrane defects, including enzyme activity assays, EMA-binding test, and osmotic gradient ectacytometry (LoRRca MaxSis). All of them showed normal values.

The third case is a male adult patient. He presented with several episodes of hemolytic crises during childhood requiring blood transfusion on two occasions. He underwent splenectomy at the age of 25-year-old. The patient was diagnosed with hereditary spherocytosis (HS) following a previous HS diagnosis of his mother and the absence of abnormal Hb peaks by conventional electrophoresis.

Patients who underwent splenectomy neither clinically improved nor presented complications as pulmonary hypertension, thrombosis or increased hemolysis during 10-year follow-up.

The last two cases are two unrelated children who presented with macrocephaly and severe congenital anemia. The parents of both patients had no family history for abnormal Hb or thalassemia and had normal hematological features. Therefore, conventional testing for abnormal Hb was not performed. All siblings were unaffected.

The first of these two unrelated children is a male patient presenting with large head circumference and hepatosplenomegaly. Congenital dyserythropoietic anemia was suspected. However, no genetic analysis was performed for confirmation. He underwent successfully bone marrow transplant at the age of 4.

The second child is a female patient presenting with frontal bossing, macrocephaly, and severe anemia at the age of 2. Congenital dyserythropoietic anemia was suspected. Therefore, genetic analysis of *CDAN1* and *SEC23B* genes was performed not revealing any disease-causing mutation. She underwent successfully bone marrow transplant when she was almost 3-year-old.

In all cases, RAD due to Hb variant was not suspected mainly due to the fact that parents did not present family history of RADs, except for case 3, RBC parameters were found to be normal and abnormal Hb fractions were absent when analyzed. Therefore, genetic testing was performed for genes associated with RADs other than globin genes, failing to show a conclusive diagnosis.

### Genetics Analysis

Written informed consent was obtained from cases or legal guardian. Targeted NGS (t-NGS) analysis was performed in cases 1, 2, and 3 while whole exome sequencing (WES) analysis was performed in cases 4 and 5. For all the patients, genomic DNA was extracted from peripheral blood. For patients who underwent bone marrow transplant, DNA samples were previously stored.

The designed t-NGS panel covered 46 genes described as disease causing for RADs, including genes responsible for membrane disorders, enzyme defects, congenital dyserytrhopoietic anemia and the *HBA1/HBA2* and *HBB* genes responsible for alpha and beta-globin chains, respectively. The full list of genes included is shown in Table 2. Exon and exon/intron boundaries were capture using a NimbleGen SeqCap EZ HyperCap (Roche) solution-based capture system followed by next generation sequencing on the MySeq (Illumina) with 150 bp paired-end reads. For the bioinformatics analysis, alignment to the hg38 genome was performed with BWA-MEM (Li H. 203 arXIV:1303.3997v2) and detection of changes with GATK^1^. Obtained variants were filtered and annotated based on variant effect, coverage (>30) and MAF (>0.05). Resulting variants were assessed for technique pitfalls through IGV. The nomenclature used was the recommended by HGVS^2^. Finally, disease-causing variants were prioritized based on inheritance pattern and VarSome^3^ for previous evidence as disease causing mutations or predictions score information. Variants were reported according to American College of Medical Genetics (ACMG) guidelines.

**TABLE 2:** List of genes included in the t-NGS approach.

Symbol	Phenotype MIM number	Gene/Locus MIM number	Category	Description
*ADA*	102700	608958	Enzymopathy	Adenosine deaminase
*AK1*	103000	103000	Enzymopathy	Adenylate kinase 1
*ALDOA*	611881	103850	Enzymopathy	Aldolase, fructose-bisphosphate a
*ANK1*	616089	612641	Membranopathy	Ankyrin 1
*ATRX*	301040	300032	Alpha-thalassemia myelodysplasia syndrome, somatic; Alpha-thalassemia/mental retardation syndrome; Mental retardation-hypotonic facies syndrome, X-linked	Helicase 2, x-linked
*BPGM*	222800	613896	Erythrocytosis and methemoglobinemia due to enzyme alteration	Bisphosphoglycerate mutase
*C15orf41*	615631	615626	Congenital dyserythropoietic anemia	Chromosome 15 open reading frame 41
*CDAN1*	224120	224120	Congenital dyserythropoietic anemia	Codanin 1
*CYB5R3*	250800	613213	Methemoglobinemia, type I; Methemoglobinemia, type II	Cytochrome b5 reductase 3
*EPB41*	611804	130500	Membranopathy	Erythrocyte membrane protein band 4.1
*EPB42*	612690	177070	Membranopathy	Erythrocyte membrane protein band 4.2
*EPO*	617907	133170	Erythropoiesis modulator	Erythropoietin
*EPOR*	133100	133171	Erythropoiesis modulator	Erythropoietin receptor
*G6PD*	300908	305900	Enzymopathy	Glucose-6-phosphate dehydrogenase
*GAPDH*	*	138400	Enzymopathy	Glyceraldehyde-3-phosphate dehydrogenase
*GATA1*	300835	305371	Congenital dyserythropoietic anemia	Gata binding protein 1 (globin transcription factor 1)
*GCLC*	230450	606857	Enzymopathy	Glutamate-cysteine ligase, catalytic subunit
*GPI*	613470	172400	Enzymopathy	Glucose-6-phosphate isomerase
*GSR*	618660	138300	Enzymopathy	Glutathione reductase
*GSS*	266130	601002	Enzymopathy	Glutathione synthetase
*GYPC*	616089	110750	Membranopathy	Glycophorin c (gerbich blood group)
*HBA1*	617981	141800	Hemoglobinopathy	Hemoglobin–alpha locus 1
*HBA2*	617981	141850	Hemoglobinopathy	Hemoglobin–alpha locus 2
*HBB*	617980	141900	Hemoglobinopathy	Hemoglobin subunit beta
*HBD*	*	142000	Thalassemia due to Hb Lepore; Thalassemia, delta-	Hemoglobin–delta locus
*HBG1*	141900	141749	Fetal hemoglobin quantitative trait locus 1	Hemoglobin, gamma a
*HBG2*	613977	142250	Cyanosis, transient neonatal; Fetal hemoglobin quantitative trait locus 1	Hemoglobin, gamma g
*HK1*	235700	142600	Enzymopathy	Hexokinase 1
*KCNN4*	616689	602754	Membranopathy	Potassium channel, calcium activated intermediate/small conductance subfamily n alpha, member 4
*KIF23*	*	605064	Congenital dyserythropoietic anemia	Kinesin family member 23
*KLF1*	613673	600599	Congenital dyserythropoietic anemia	Kruppel-like factor 1 (erythroid)
*NT5C3A*	266120	606224	Enzymopathy	5′-nucleotidase, cytosolic iiia
*PFKL*	*	171860	Hemolytic anemia due to phosphofructokinase deficiency	Phosphofructokinase, liver type
*PFKM*	232800	610681	Enzymopathy	Phosphofructokinase, muscle
*PGD*	*	172200	Enzymopathy	6-phosphogluconate dehydrogenase, erythrocyte
*PGK1*	300653	311800	Enzymopathy	Phosphoglycerate kinase 1
*PIEZO1*	616089	611184	Membranopathy	Piezo-type mechanosensitive ion channel component 1
*PKLR*	266200	609712	Enzymopathy	Pyruvate kinase, liver and rbc
*RHAG*	185000	180297	Membranopathy	rh-associated glycoprotein
*SEC23B*	224100	610512	Congenital dyserythropoietic anemia	Sec23 homolog b, copii coat complex component
*SLC2A1*	606777	138140	Membranopathy	Solute carrier family 2 (facilitated glucose transporter), member 1
*SLC4A1*	612653	109270	Membranopathy	Solute carrier family 4 (anion exchanger), member 1 (diego blood group)
*SPTA1*	130600	182860	Membranopathy	Spectrin alpha, erythrocytic 1
*SPTB*	616649	182870	Membranopathy	Spectrin beta, erythrocytic
*TPI1*	615512	190450	Enzymopathy	Triosephosphate isomerase 1
*UGT1A1*	237900	191740	Gilbert syndrome	udp glucuronosyltransferase 1 family, polypeptide a1

*Not available.

For case 4, WES was performed in a trio approach (patient and both parents). Libraries were prepared using the Kapa HTP kit (Illumina, San Diego, CA, United States) and capture was performed using the SeqCap EZ Human Exome Library v3.0 (Roche NimbleGen Madison, WI, United States). Sequencing was done on an Illumina HiSeq2500 HTv4 (Illumina, San Diego, CA, United States) with paired-end 125-bp reads. Read alignment to hg19 and variant calling were done with a pipeline based on BWA-MEM0.7 and GATK 3.3.0. The median coverage of the captured target region was at least 98×. Variant annotation and prioritizing were done using Cartagenia Bench Lab NGS (Agilent Technologies). Variants located outside the exons and intron/exon boundaries and variants with a minor allele frequency (MAF) of >1% in control databases, including dbSNP137^4^, 1000 Genomes Project (phase 3)^5^, and Exome Variant Server (EVS), NHLBI Exome Sequencing Project National Heart, Lung, and Blood Institute GO Exome Sequencing Project (ESP6500 release)^6^ and in-house exome controls were excluded. Variants that fitted with a *de novo* or recessive mode of inheritance were further prioritized based on literature, predicted (deleterious) effects on protein function by e.g., truncating the protein, affecting splicing, amino acid change, and evolutionary conservation.

For case 5, WES was performed in a trio approach (patient and both parents). Exomes were captured using the Agilent SureSelectXT Human All Exon v5 (Agilent, Santa Clara, CA, United States) accompanied by Illumina paired-end sequencing on the HiSeq2000 (Illumina, San Diego, CA, United States). The in-house sequence analysis pipeline Modular GATK-Based Variant Calling Pipeline (MAGPIE) (LUMC Sequencing Analysis Support Core, LUMC) was used to call the SNVs/indels. LOVDplus (Leiden Genome Technology Center, LUMC, Leiden) was used for interpretation of variants.

## Results

Genetic variants in globin genes responsible for UH or HUH were found in all five cases as shown in Table 1. All variants were confirmed by Sanger sequencing.

In case 1, variant *HBB*:c.202G > A (p.Val67Met) was found in exon 2 in the heterozygous state. This *HBB* variant is known as Hb Bristol-Alesha, a UH associated with moderate-severe hemolytic anemia. The variant was not found in the parents, suggesting a *de novo* variant in the patient.

In case 2, variant *HBA1*:c.187G > A (p.Val62Met) was found in exon 2 in the heterozygous state. This *HBA1* variant is known as Hb Evans and is associated wild with mild hemolytic anemia and classified as UH. Parents were not sequenced.

In Case 3, variant *HBB*:c.290T > C (p.Leu96Pro) was found in in exon 2 in the heterozygous state. This *HBB* variant is known as Hb Debrousse and is described as a moderate UH. Parents were not sequenced. Nevertheless, antecedents of hemolytic anemia are present in the mother, suggesting a dominant inheritance pattern.

In cases 4 and 5, two missense stop-loss mutations at position 422 of the *HBB* gene were found. The first variant *HBB:*c.442T > C (p.Ter147Glnext^∗^21), found in case 4, is known as Hb Zunyi, while the second variant *HBB:*c.442T > A (p.Ter147Lysnext^∗^21), found in case 5, constitutes a novel variant which was called Hb Mokum. Both variants cause the loss of a stop codon and elongation of the translated beta-globin chain of 21 amino acids due to a new stop codon in the 3′ untranslated region (3′UTR) of the *HBB* gene. The variants were not found in the parents suggesting *de novo* variants in the patients.

According to the ACMG guidelines, all the variants were classified as pathogenic (Richards et al., 2015).

## Discussion

We highlight the importance of including globin genes in the NGS analysis of RAD for enabling the diagnosis of UH. We present five clinical cases affected with RAD due to UH variants, four are related to the beta-globin gene (Hb Bristol—Alesha, Hb Debrousse, Hb Zunyi, and the novel Hb Mokum), meanwhile, one is related to the alpha-globin gene (Hb Evans). The use of NGS has been crucial for the final conclusive diagnosis.

The severity of RADs due to UHs depends on the mutation’s impact on protein stability and consequently on the degree of hemolysis and inefficient erythropoiesis. Patients’ RBCs typically display abnormal but unspecific morphology with microcytosis, hypochromia, moderate to severe anisopoikilocytosis, basophilic stippling, and inclusions that may become particularly prominent following splenectomy (Steinberg et al., 2009; Kent et al., 2014). UHs are commonly inherited in a dominant way or presented as *de novo*, although there are some examples of recessive inheritance leading to mild phenotypes. According to results obtained through the HbVar Query page (dated 14th January 2020), 1,534 Hb variants have been described so far due to mutations on either *HBA1*/*HBA2* or the *HBB* genes. Up to 251 variants (16.4%) are classified as UH or HUH based on heat or isopropanol stability tests and/or low Hb abundancy (Giardine et al., 2007, 2014). It is worthy to highlight that all the HUH variants involving the *HBB* gene reported positive stability tests, meanwhile in most of the HUH involving the alpha-globin genes, hyper instability has been only deduced from low abundance. This must be cautiously taken since mutations in alpha-globin genes are lower expressed (<25%) than in beta-globin gene due to the existence of duplicated alpha-globin genes, *HBA1* and *HBA2*, especially in mutations involving the *HBA2* gene, as it encodes a 2-3–fold higher level of mRNA than *HBA1* (Liebhaberts et al., 1986). Thus, the beta-globin gene is the first option to investigate for disease-causing mutations leading to RADs due to UHs/HUHs especially in cases with moderate to severe phenotypes.

Interestingly, Hb Bristol-Alesha is classified as a UH variant, not as a HUH as we expected based on the severity of the patient’s clinical picture. The change to methionine at position 67 of the beta-globin chain alters the hydrophobic heme pocket causing the instability of the protein (Kano et al., 2004). As described in previous clinical reports, at physical examination, splenomegaly and jaundice may be found. Iron overload and gallstones may develop due to the rapid turnover of RBCs.

Hb Debrousse, reported twice in literature, is a UH characterized by well-compensated chronic hemolytic anemia due to its high oxygen affinity. Hb Debrousse is caused by leucine to proline substitution at position 96 involving the hydrophobic environment of the proximal side of the heme. In the previously reported cases, Hb Debrousse discovery was possible after a Parvovirus B19 infection that caused a hemolytic crisis (Lacan et al., 1996). Indeed, since affected patients show a chronic well-compensated hemolytic anemia, the diagnosis of such a variant is unlikely until the globin genes are investigated. Such a study is usually performed only when some complications occur.

Hb Zunyi was recently reported for the first time as a *de novo* mutation in a Chinese child with severe anemia requiring blood transfusion, malnutrition, growth delay, splenomegaly and hepatomegaly (Su et al., 2019). In the study herein, we identified both Hb Zunyi and Hb Mokum as *de novo* mutations in the heterozygous state. Hb Zunyi and the novel Hb Mokum are stop-loss mutations at position 442 in *HBB*, resulting in an elongated beta-globin chain leading to HUHs. The extra amino acids in the elongated beta-globin chain (169 a.a.) are probably affecting its helical sequence, interfering with its tertiary structure and causing an unstable tetramer. Frameshift mutations in the *HBB* gene, resulting in the elongated beta-globin chain, have been described before but resulted in shorter beta-chains (max. 157 aa.) and milder phenotypes than the mutations described here (Su et al., 2019).

Finally, Hb Evans is classified as UH. It is consequence of a valine to methionine substitution at position 62 of the alpha2-globin chain encoding gene *HBA2*. Hb Evans has been reported in patients presenting with mild hemolytic anemia that was getting worse particularly in case of stress (Wilson et al., 1989).

The standard tests to detect abnormal anemias are High Precision Liquid Chromatography (HPLC) or conventional or capillary electrophoresis (CE). However, UHs/HUHs do not normally appear in the peak-patterns or appear as small peaks that may be mistaken for degradation products. In three of the five UH cases reported here, extra peaks were not detected. More confined methods are Heinz Bodies test or stability tests as isopropanol precipitation or heat stability tests, which are affordable screening techniques for UHs/HUHs variants. In the patient with Hb Bristol-Alesha, Heinz bodies were detected and heat stability test was positive only after splenectomy (Figures 1, 2), while in the other patients, Heinz bodies and heat stability test were not performed. Genetic analysis of globin genes should be performed for diagnosis confirmation. Inclusion of *HBA1/HBA2* and *HBB* in NGS approaches will facilitate timely conclusive diagnosis. as a screening tool for hemolytic anemias will assist in reaching a definitive diagnosis sooner.

Furthermore, the occurrence of *de novo* mutations causing UHs/HUHs should also be considered in the analysis of genetic variants.

The usefulness of NGS in improving the diagnosis of RADs has already been demonstrated in several studies as well as its relevance in new gene discovery (Shang et al., 2017; Duez et al., 2018). In the case of overlapping phenotypes, which frustrate proper diagnosis, the use of NGS may be beneficial for ultra-rare RADs. In a recent publication, 36% of patients initially diagnosed with congenital dyserytrhopoietic anemia, received a final diagnosis of pyruvate kinase deficiency after NGS analysis (Russo et al., 2018). Nevertheless, in the majority of the t-NGS panels reported, globin genes are not included, since globin genes are quite short and molecular diagnosis of most common Hb disorders, such as sickle cell disease (SCD) and thalassemia syndromes, is well-established through Sanger sequencing and GAP-PCR/MLPA. Therefore, dBTHAL disorders due to UH/HUH may also benefit from NGS approaches for RADs by including globin genes, as presented herein.

Current literature on dBTHAL and UH/HUH variants is mainly composed of retrospective case reports, which makes evidenced-based management of this RAD unlikely. In addition, to benefit from the most adequate management it is necessary to achieve a diagnosis as early as possible. In conclusion, this study confirms the importance of NGS as a fundamental tool to early identify and treat UH/HUH in patients with RAD without an established diagnosis after standard methodologies.

Future challenges include a better understanding of disease characteristics and management, and consideration of bone marrow transplant as a curative option. Therefore, we encourage that these patients are referred to expert Units in referral centers for enabling basic and clinical research taking advantage of the already established European Reference Networks for rare hematological disorders, ERN-EuroBloodNet.

## Data Availability Statement

The datasets generated for this study can be found in the online repositories. The names of the repository/repositories and accession number(s) can be found below: www.ithanet.eu and https://ithanet.eu/db/ithagenes?ithaID=3697.

## Ethics Statement

Written informed consent was obtained from the cases/legal guardian for the publication of any potentially identifiable images or data included in this article.

## Author Contributions

VR, MM-P, CLH, TK, and DB wrote the manuscript. All authors critically revised the manuscript.

## Conflict of Interest

The authors declare that the research was conducted in the absence of any commercial or financial relationships that could be construed as a potential conflict of interest.

## Footnotes

1 https://software.broadinstitute.org/gatk/

2 http://www.hgvs.org

3 https://varsome.com

4 http://www.ncbi.nlm.nih.gov/projects/SNP

5 http://www.internationalgenome.org/

6 http://evs.gs.washington.edu/EVS/
